# Time-Resolved Photoelectron Spectroscopy of Conical Intersections with Attosecond Pulse Trains

**DOI:** 10.1021/acs.jpclett.1c01843

**Published:** 2021-08-19

**Authors:** Deependra Jadoun, Markus Kowalewski

**Affiliations:** Department of Physics, Stockholm University, Albanova University Centre, SE-106 91 Stockholm, Sweden

## Abstract

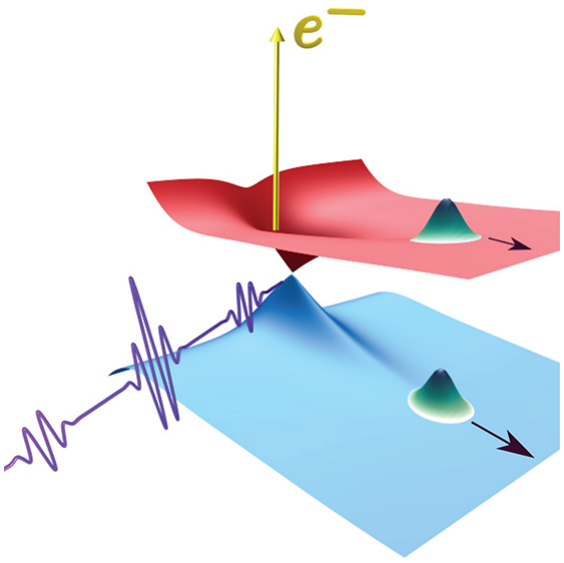

Conical Intersections (CIs), which are believed to be ubiquitous in molecular and biological systems, open up ultrafast nonradiative decay channels. A superposition of electronic states is created when a molecule passes through a CI and the nuclear wave packet branches. The resulting electronic coherence can be considered a unique signature of the CI. The involved electronic states can be resolved in the energy domain with photoelectron spectroscopy using a femtosecond pulse as a probe. However, the observation of the created electronic coherence in the time domain requires probe pulses with several electron volts of bandwidth. Attosecond pulses can probe the electronic coherence but are unable to resolve the involved electronic states. In this Letter, we propose to address this restriction by using time-resolved photoelectron spectroscopy with an attosecond pulse train as a probe. We theoretically demonstrate that the resulting photoelectron spectrum may yield energy resolution as well as the information on the created coherences in the time domain.

Conical intersections (CIs)^1−4^ are known to play a crucial role in many photochemical and photobiological processes, such as photosynthesis,^5^ photoisomerization in the process of vision,^6^ and ultraviolet (UV) light induced damage in DNA.^7^ CIs form in a molecule when two or more electronic states become degenerate, and the electronic and nuclear degrees of freedom are strongly coupled. In the vicinity of a CI, the Born–Oppenheimer approximation breaks down and opens up ultrafast nonradiative decay channels, funneling the molecule back to the ground state. The appearance of CIs in a molecule results in population transfer, a rapid change of properties such as transition dipole-moments, and the generation of electronic coherence due to the branching of the nuclear wave packets. CIs mediate many important photochemical processes, but the direct observation of the rapidly vanishing separation between the electronic states in the vicinity of CI is still challenging. Recent developments in X-ray science and ultrafast physics have opened up the possibility to observe the passage through a CI with increasing accuracy.^8−18^ Several X-ray-based time-resolved Raman spectroscopic techniques have been proposed to observe the electronic coherence generated near a CI.^19−21^ Transient absorption spectroscopy has been very successful for the detection of CIs in molecules.^22−28^ Spontaneous X-ray emission spectroscopy^28−30^ as well as X-ray diffraction^31^ may provide complementary information about the nonadiabatic dynamics of molecules.

Recent progress in the generation of attosecond pulses allows for the observation of electron dynamics,^32−39^ which may also prove useful for more accurate measurements with respect to the CI induced nuclear-electronic dynamics. The direct observation of the rapidly evolving energy gap in the vicinity of a CI requires a bandwidth of several electronvolts, which can be provided by attosecond pulses. The high-harmonic generation (HHG) process^40^ is commonly used to generate attosecond pulses. However, HHG directly produces attosecond pulse trains (APTs) not isolated attosecond pulses. APTs have been used, for example, in pump–probe experiments to observe the electron wave packet in nitrogen molecules.^41^ It has been demonstrated that APTs can monitor and manipulate the nuclear wave packets in deuterium molecules, thus giving control over ionization and dissociation pathways.^42^ We propose to perform time-resolved photoelectron spectroscopy (TRPES) with an APT as a probe–pulse in order to resolve a CI in a molecular system. It has previously been proposed that TRPES^43^ with a single attosecond pulse as a probe may be capable of detecting the occurrence of CI in a system. Using single attosecond pulses (SAPs) as a probe in TRPES can facilitate the observation of the electronic coherence, which originates from the superposition of electronic states and is generated in the vicinity of a CI. The frequency of the coherent oscillations appearing in the time-dependent signal corresponds to the energetic separation between the two involved valence states. However, this scheme comes with the restriction that the electronic states involved in the process cannot be resolved in the energy domain. A spectrally narrow probe pulse is required to resolve the two electronic states involved in the CI. Using spectrally narrow pulses results in a loss of the coherent oscillation in the time domain due to a vanishing overlap between the photoelectrons originating from contributing electronic states. In this letter, we show how using APTs as a probe in photoelectron spectroscopy may bypass this limitation.

With TRPES,^44−54^ the molecule is first prepared to a nonstationary valence state using an UV pump pulse. The dynamics of the prepared system is then probed by an ionizing probe–pulse, generating the photoelectrons. The kinetic energy of the photoelectrons, which is the difference between the energy of the ionizing pulse and the ionization potential, is recorded to construct the time-resolved spectrum. The TRPES signal is defined as the integrated photoelectron current and can be derived via time-dependent perturbation theory.^43,55^ It can then be expressed as a function of pump–probe delay *T* and the photoelectron energy ω_*p*_:
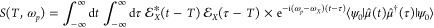
1where  is electric field corresponding to the ionizing pulse with ω_*X*_ being its center frequency, μ̂ is the dipole operator corresponding to the ionization process, and |ψ_0_⟩ is the wave function in the valence states. Note that the transition dipole moment μ is considered to have a constant value in our model of study. However, it is possible to use, for example, Dyson orbitals^56−58^ to calculate transition dipole moments for a more accurate description of the ionization process. The details of the quantum dynamics simulations, the Hamiltonian, and the model parameters used for simulations can be found in the Supporting Information (SI).

The APT is obtained by the HHG process. The HHG process is usually driven by a short infrared (IR) pulse, which interacts with the atomic gas. The ionization and recombination of the electrons from the parent ion yield the high-harmonic spectra. We define the APT as an infinite train of attosecond pulses enclosed in a Gaussian envelope, which can be expressed as follows:^60,61^
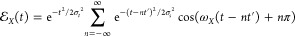
2where *t*′ = π/ω_*I*_ with ω_*I*_ being the frequency of the IR pulse that initiates the HHG process, σ_*t*_ is the envelope of the APT, σ_*s*_ is the envelope of an attosecond burst, and ω_*X*_ is the center frequency of the attosecond bursts in the ionizing APT. The center frequency of the IR field is assumed to be filtered out and does not interact with the sample.

Each attosecond burst of the APT covers the same frequency range in the spectral domain. All attosecond bursts have a fixed phase relationship with respect to each other. The attosecond bursts interfere with each other in the spectral domain, thus generating various peaks at odd harmonics with respect to the IR driving pulse. The number of attosecond bursts in the system and the amplitude of each burst is determined by the overall pulse width of the APT (eq 2). Thus, σ_*t*_ indirectly controls the width of each harmonic in the spectral domain. The shape of the APT in the spectral domain can be understood as a convolution between each attosecond burst and the Gaussian envelope of the APT.^61^

The basic principle of TRPES with APTs is demonstrated for a two-level system in the scheme shown in Figure 1. Here we assume an APT with three harmonics, which creates three photoelectron peaks per state. If the separation between the harmonics (2ω_*I*_) is resonant with the separation between the states |*a*⟩ and |*b*⟩, peaks originating from different harmonics ionizing different states overlap with each other. The peak of harmonic *H*_47_, for example, ionizing state |*a*⟩ overlaps with the peak corresponding to *H*_45_ ionizing state |*b*⟩ (see Figure 1). When |*a*⟩ and |*b*⟩ are initially in a superposition, the overlapping peaks will exhibit an oscillation pattern with frequency ω_*b*_ – ω_*a*_. The presence of this oscillation pattern can thus be interpreted as a signature of an electronic coherence.

**Figure 1:**
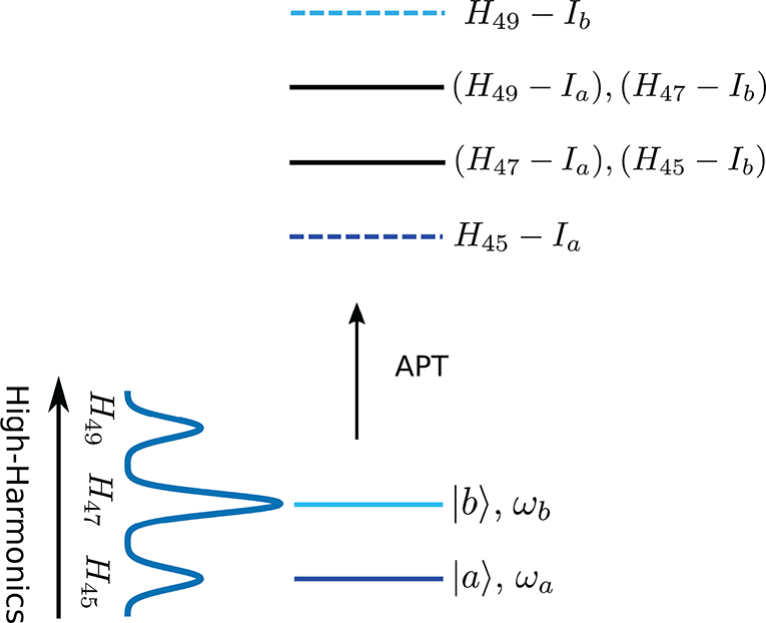
Schematics of the ionization process with an APT. The final ionized state is the same for both the initial states |*a*⟩ and |*b*⟩, and *I*_*a*_ (*I*_*b*_) is the ionization potential for state |*a*⟩ (|*b*⟩). The black lines represent the overlap of two signals corresponding to different harmonics and states. The dashed lines correspond to the photoelectron signal that does not overlap with any other signal.

A molecular model system based on the dissociation dynamics of a molecule is used to demonstrate the idea. The 1D slices of the diabatic PESs of the model are shown in Figure 2(a). The analytical expressions used to construct 2D diabatic PESs and the diabatic couplings between the valence states can be found in the SI. Note that the diabatic couplings are chosen such that the diabatic-to-adiabatic transformation generates two valence states that exhibit a CI. A close-up of the CI between the valence states in the adiabatic basis is shown in Figure 2(b). The molecule is assumed to be excited by an actinic pump pulse, which launches the dynamics in the *V*_1_ state. To optimize the spectrum the pump-pulse should be kept short enough to avoid any significant nuclear motion within its duration. A nuclear wave packet, which has been stretched out by long pump-pulse would decrease the modulation depth of the coherent oscillations in the signal. The variation of the population in each state as a function of time delay is shown in Figure 3(c). When the wave packet reaches the diabatic coupling region at ≈5 fs, the diabatic population transfer takes place between the valence states *V*_1_ and *V*_0_ and creates an electronic coherence.

**Figure 2:**
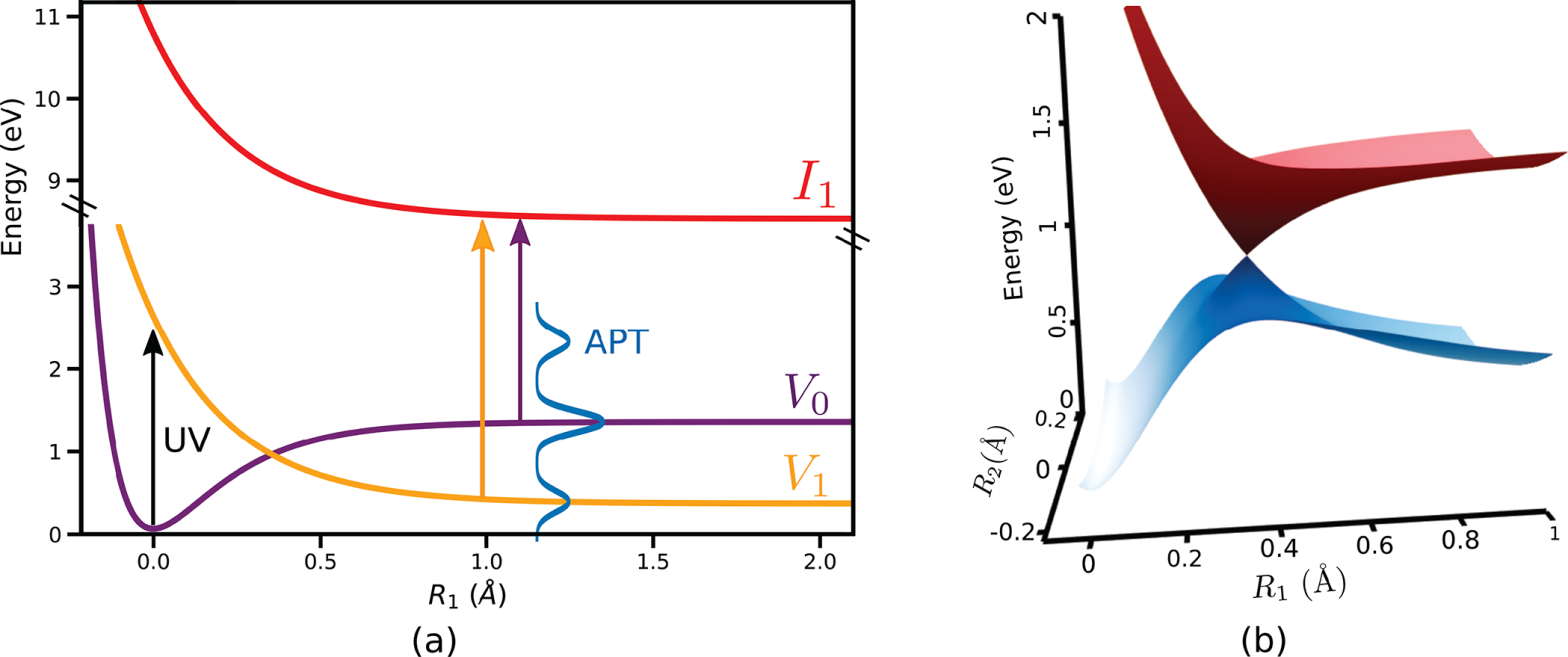
(a) 1D slices of the diabatic PESs for the ground state *V*_0_ (purple), the excited state *V*_1_ (orange), and the ionized state *I*_1_ (red). The variation of the energy along the reaction coordinate *R*_1_ is shown. (b) CI between the valence states in the adiabatic representation.

**Figure 3:**
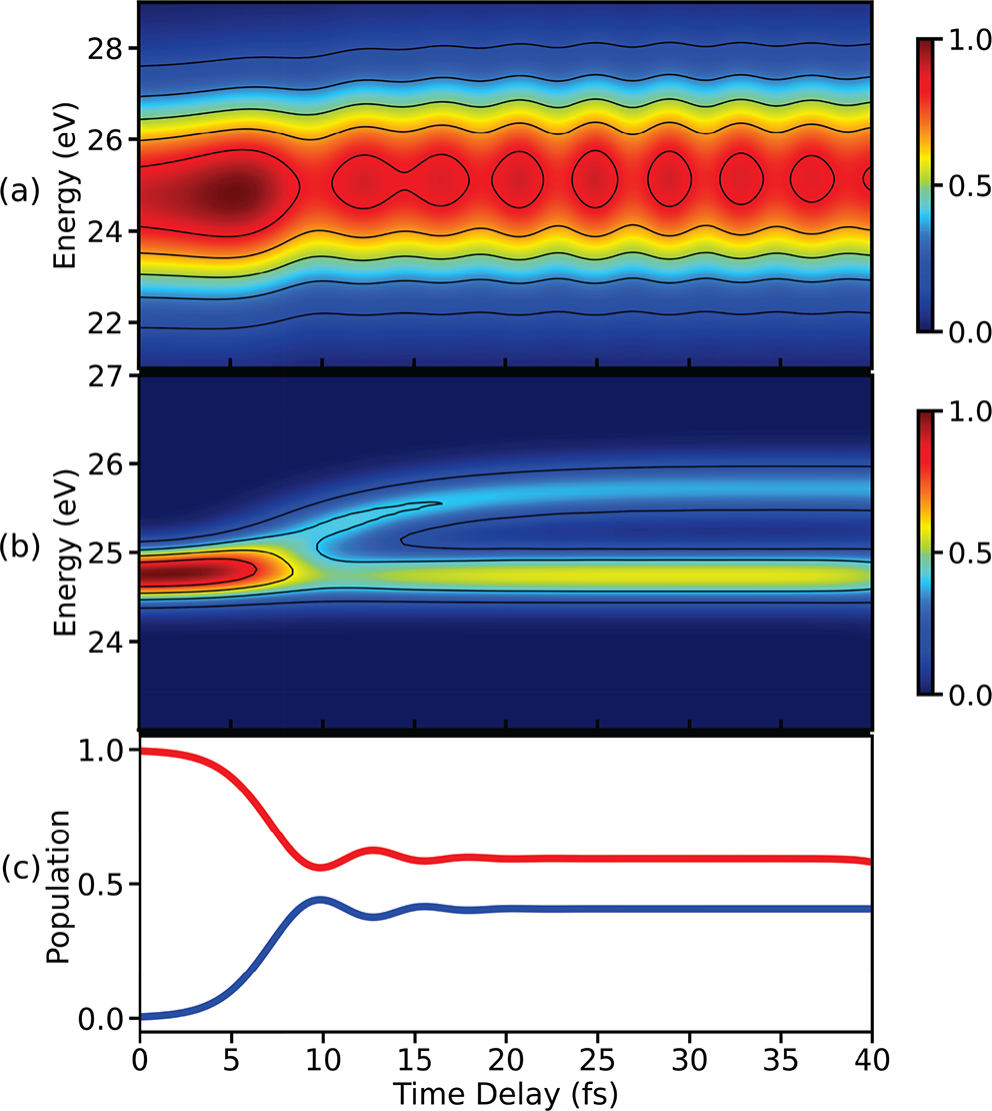
TRPES spectra for single pulses with pulse widths (fwhm) (a) 0.71 fs and (b) 5.29 fs are shown in this figure. Both the ionization pulses have a center frequency of 32.9 eV. The electronic coherence generated in the molecule after the passage through the CI is resolved in (a) due to use of ultrashort pulse. On the other hand, the spectral resolution is achieved in (b) when a few-femtosecond pulse is used for ionization. (c) Variation of population in states *V*_0_ (blue) and *V*_1_ (red) as a function of time delay is shown here. The population in state *V*_0_ starts to increase around 5 fs, indicating the arrival of wave packets in the vicinity of CI.

First, we show the TRPES spectra (see eq 1) calculated using single attosecond and femtosecond pulses and then compare those with the spectra calculated using APT. The TRPES signals for two single pulses with different pulse widths are shown in Figure 3. Figure 3(a) shows the TRPES signal constructed using an ultrashort ionizing pulse with a full width at half maximum (fwhm) of 0.71 fs. The signal shows the generation of electronic coherences after the passage through the CI around 10 fs: the broad spectral width of the ionizing pulse creates a single, broad photoelectron peak in the spectral domain, allowing for the signal from two electronic states to overlap. The overlap results in an oscillating interference pattern caused by the presence of electronic coherence in the system.

Figure 3(b) shows the spectrum constructed using a few-femtosecond ionizing pulse with fwhm of 5.29 fs. Two peaks corresponding to the two electronic states appear after 10 fs indicating the branching of wave packets in the vicinity of CI. The shape of the two electronic states involved in the CI can be observed in the energy domain. The separation between the two electronic states after 20 fs remains constant at ≈1 eV. Since the applied pulse is narrow in the spectral domain, there is a vanishing overlap between the photoelectron signals originating from the two electronic states, and interference does not occur. The photoelectron yield in the spectral domain is related to the Fourier transform of the ionizing pulse. This results in a trade-off between the spectral resolution and the temporal resolution that a pulse can provide. Hence, resolving both electronic coherence and the electronic states at the same time, only using a single pulse is not possible.

When constructing the photoelectron signal using APT as an ionizing pulse, the 47^*th*^ harmonic of the IR driving field is considered to be the center frequency of each attosecond burst in the APT. The signals calculated using an APT are shown in Figure 4. Three different IR fundamental frequencies are used to generate TRPES spectra. The three strongest peaks of the spectrum constructed with an APT, using ω_*I*_ = 0.5 eV, are shown in Figure 4(a). The branching of wave packets due to the CI becomes visible between 10 and 15 fs, indicating that the involved states can be resolved. Note that the width of the single photoelectron peaks, which allows for the resolution in the energy domain, are determined by σ_*t*_. The oscillations caused by the electronic coherence start appearing in each peak around 15 fs. Since the harmonics in this case are separated by 1 eV, the overlap between the signal from two electronic states results in the interference after 15 fs, thus indicating the presence of the electronic coherence in the system.

**Figure 4:**
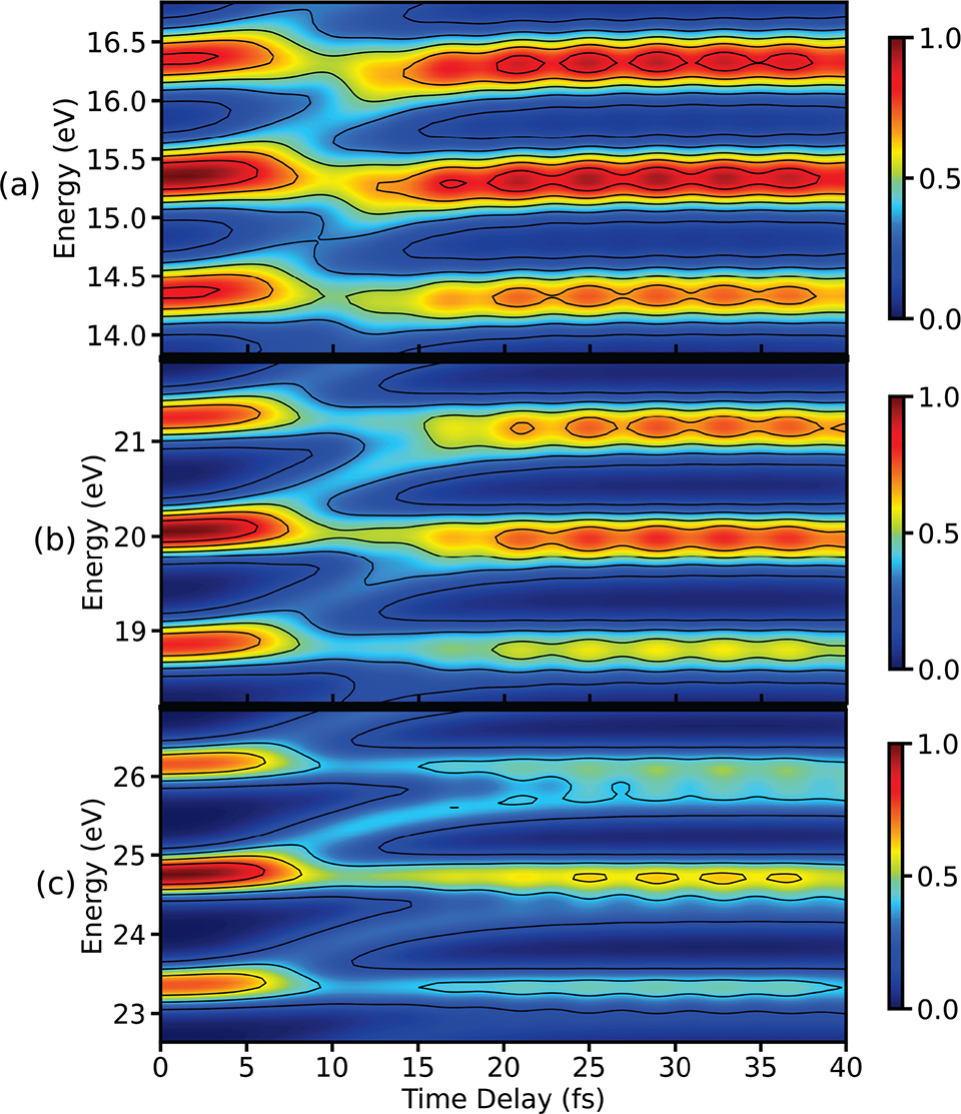
Three most intense peaks in the TRPES signal for an APT with (a) ω_*I*_ = 0.5 eV, (b) ω_*I*_ = 0.6 eV, and (c) ω_*I*_ = 0.7 eV are shown here. The other parameters of the APT are σ_*t*_ = 2.55 fs (fwhm = 6 fs) and σ_*s*_ = 0.25 fs (fwhm = 0.59 fs).

Next, the frequency of the IR driving pulse is increased to 0.6 eV (Figure 4(b)) and the splitting between peaks increases accordingly. The signature of the branching after the CI is now represented in the photolectron energy, as it appears from 10 to 20 fs. The interference pattern originating from electronic coherence appears after ≈20 fs and is weaker compared to signal shown in Figure 4(a) for ω_*I*_ = 0.5 eV. With the increased separation between the harmonics, its resonance with the electronic states decreases. This results in a reduced overlap between the two signals and hence the interference is also decreased. When the harmonics are not in optimal resonance with the two electronic states, the strength of interference solely depends on the width of each harmonic in spectral domain. A similar behavior is observed in the spectrum calculated for an APT with ω_*I*_ = 0.7 eV which is shown in Figure 4(c). The electronic states are completely visible in this case, and a weak interference pattern appears after ≈25 fs, indicating the presence of electronic coherence.

The model has been constructed to obtain a long-living electronic coherence, but in a molecular system the gradients of the involved states may differ significantly. The electronic coherence would be subject to rapid decay. To resolve the electronic coherence in such a scenario, the photoelectron side bands need to overlap in the spectrum before the electronic coherence decays. Hence, the time window for the observation of electronic coherence will be shorter in a molecule with rapidly varying energy gap. Note that the underlying principle is analogous to the RABBITT^40,62^ technique in which the quantum interference is created by the side bands of an IR streaking field. In this study, no IR streaking field was required to generate the interference pattern, but rather the harmonics themselves are responsible for the interference.

In conclusion, we have simulated the photoelectron signal for a system which contains a CI, by using an APT as a probe–pulse. Our simulations show that the interference between two neighboring harmonics can be used to selectively detect the electronic coherence created by the CI. The only requirement is that the ionization from two different harmonics overlaps in the ionic state and coincides with the energy difference of the involved electronic states. The energy resolution is preserved in this scheme and increasing energy gap, after the passage through the CI, can be observed in the spectrum. Note that with a single Gaussian probe pulse, the energy resolution has to be traded for time resolution to observe the coherent oscillation caused by the electronic superposition.^43^ The time-energy uncertainty principle imposed by the linear probe could thus be bypassed by using an APT.

## Supporting Information Available

Model system including PES, TRPES signal including diabatic coupling terms and loop diagrams, quantum dynamics simulations, and TRPES specturm (closeup of Figure 4b) (PDF)
