# Supplementary material for: Time-Resolved Photoelectron Spectroscopy of Conical Intersections with Attosecond Pulse Trains
Source: J Phys Chem Lett. 2021 Aug 19;12(33):8103–8. doi: 10.1021/acs.jpclett.1c01843 (PMC8404190; doi:10.1021/acs.jpclett.1c01843)
Supplement: Supplementary file 1 — jz1c01843_si_001.pdf [file jz1c01843_si_001.pdf]

Supporting Information: Time-resolved Photoelectron Spectroscopy of Conical Intersections with Attosecond Pulse Trains

Deependra Jadoun and Markus Kowalewski*

Department of Physics, Stockholm University,

Albanova University Centre, SE-106 91 Stockholm, Sweden

I. MODEL SYSTEM

We use atomic units ($\hbar = e = m_e = 4\pi\epsilon_0 = 1$) throughout the rest of the document unless stated otherwise. The Hamiltonian of the molecular system is defined as follows,

$$\hat{H}_0 = \begin{bmatrix} \hat{T} + \hat{V}_0 & \hat{C}_{01} & 0 \\ \hat{C}_{01} & \hat{T} + \hat{V}_1 & 0 \\ 0 & 0 & \hat{T} + \hat{I}_1 \end{bmatrix} \tag{S1}$$

where $\hat{T} = (-1/2m_r)\nabla^2$ is the kinetic energy operator with ∇ being the gradient operator with respect to the reaction coordinates R_1 and R_2, m_r represents the reduced mass, $\hat{V}_0$ and $\hat{V}_1$ represent the potential energy surfaces (PESs) for the two valence states with $\hat{I}_1$ being the ionic state, and $\hat{C}_{01}$ represent the diabatic couplings between the valence states. The operators $\hat{V}$, $\hat{I}$ and $\hat{C}_{01}$ depend on the two reaction coordinates R_1 and R_2. The diabatic potential energy surfaces and the diabatic coupling terms were constructed as a function of reaction co-ordinates R_1 and R_2 using the following expressions,

$$\begin{aligned} V_0 &= (0.2 + 2e^{-6(R_1-0.1)} - e^{-2.5(R_1-0.1)})/4 + R_2^2/36 \\ V_1 &= (0.05 + 2e^{-2R_1})/4 + (R_2 - 0.2)^2/36 \\ C_g &= 0.001e^{-2(R_1-1.206)^2}(1 - e^{-5|R_2|})\left(1 - \left|\frac{R_2 - |R_2|}{R_2}\right|\right) \\ C_{01} &= C_g/(80 \times \max(C_g)) \\ I_1 &= V_1 + 0.3 \end{aligned} \tag{S2}$$

* E-mail: markus.kowalewski@fysik.su.se

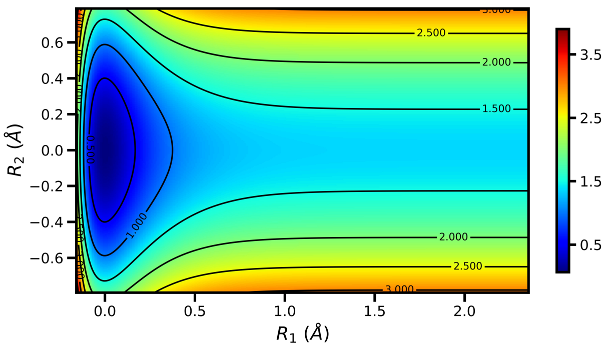

Figure S1. The PES for the valence state V_0 is shown here. The energy (in eV) is represented on the color bar.

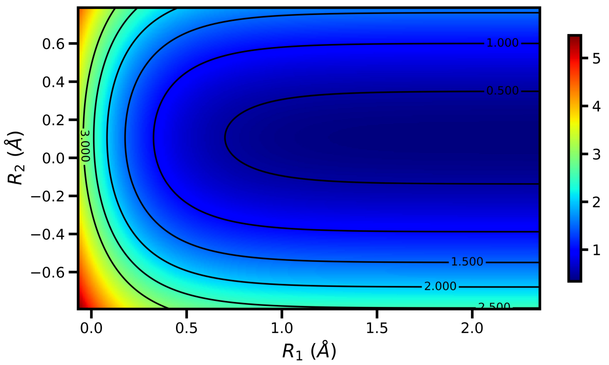

Figure S2. The PES for the valence state V_1 is shown here. The energy (in eV) is represented on the color bar.

The PES of the V_0 state is shown in Fig. S1, the PES of the V_1 state is shown in Fig. S2, and the diabatic couplings are shown in Fig. S3.

II. TRPES SIGNAL

The total Hamiltonian of the system in the presence of the ionizing probe-pulse is defined as follows [1],

$$\hat{H}(t) = \hat{H}_0 + \hat{H}_{int}(t) + \hat{H}_p \tag{S3}$$

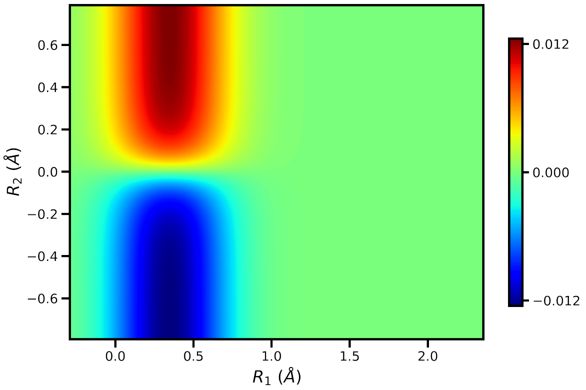

Figure S3. The diabatic coupling terms between the valence states V_0 and V_1 are shown here.

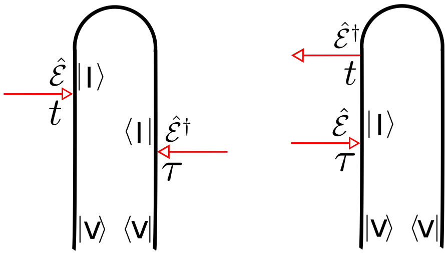

Figure S4. Loop diagrams corresponding to the TRPES signal[2] are shown here. The time runs along the loop from left bottom to right bottom. The interaction from left acts on the ket, and from the right acts on the bra. The arrows pointing inwards represent the absorption of the photon. $|\mathrm{V}\rangle$ represents the superposition of valence states V_0 and V_1, and $|\mathrm{I}\rangle$ represents the ionized states.

where $\hat{H}_0$ is the molecular Hamiltonian defined in Eq. S1, $\hat{H}_{int}$ is the interaction Hamiltonian representing the interaction between the molecule and ionizing pulse, and $\hat{H}_p = \sum_p \omega_p \hat{c}_p^\dagger \hat{c}_p$ represents the Hamiltonian corresponding to the photoelectron with $\hat{c}_p(\hat{c}_p^\dagger)$ being the annihilation (creation) operator for photoelectrons with energy ω_p. The time-dependent interaction Hamiltonian can be defined by,

$$\hat{H}_{int}(t) = \left(\hat{\mathcal{E}}_X(t) + \hat{\mathcal{E}}_X^\dagger(t)\right) \times \left(\hat{\mu}_p(t) + \hat{\mu}_p^\dagger(t)\right) \tag{S4}$$

where $\hat{\mu}_p(\hat{\mu}_p^\dagger)$ is the photoelectron annihilation (creation) operator, and $\hat{\mathcal{E}}_X$ represents the electric field operator related to the ionizing pulse. The photoelectron signal is defined as the integrated rate

of change of the number of photoelectrons and can be written as,

$$S(T,\omega_p) = \int_{-\infty}^{\infty} \mathrm{d}t \frac{\mathrm{d}\left\langle \hat{N}_p \right\rangle}{\mathrm{d}t} \tag{S5}$$

where $\hat{N}_p = \hat{c}_p^\dagger \hat{c}_p$ is the photoelectron number operator. The above equation is solved by treating the interaction between the ionizing pulse and the molecule as a perturbation. The signal expression, which can be read off the loop diagrams in Fig. S4, is given by[1],

$$S(T,\omega_p) = \int_{-\infty}^{\infty} \mathrm{d}t \int_{-\infty}^{\infty} \mathrm{d}\tau \mathcal{E}_X^*(t-T)\mathcal{E}_X(\tau-T) e^{-i(\omega_p-\omega_X)(t-\tau)} \langle\psi_0| \hat{\mu}(t)\hat{\mu}^\dagger(\tau) |\psi_0\rangle \tag{S6}$$

where $\mathcal{E}_X$ is electric field corresponding to the ionizing pulse with ω_X being its center frequency, μ is the dipole operator corresponding to the ionization process, and $|\psi_0\rangle$=$(|\phi_0\rangle, |\phi_1\rangle)^T$ with $|\phi_0\rangle$ and $|\phi_1\rangle$ being the nuclear wave-functions in the V_0 and V_1 states, respectively. Note, that the transition dipole-moments for both the valence states are considered the same in our study. When the transition dipole-moments for the two valence states are not comparable, the amplitude of the coherent oscillations in the time-resolved signal scales linearly with the ratio of the transition dipole moments.

III. QUANTUM DYNAMICS SIMULATIONS

The direct propagation simulation protocol[3] is used to calculate the correlation function in Eq. S6. Our in-house software QDng is used to simulate the wave-packet dynamics using Arnoldi method[4]. The PESs and the wave functions are represented on a numerical grid with dimension 300×300. The second order derivative with respect to the reaction coordinates in the kinetic energy operator is calculated using the Fourier transform method. The step-size of time for the wave packet propagation is 48.4 attosecond. The reduced mass m_r used in the kinetic energy term is 30,000. A perfectly matched layer[5] is used to absorb the wave-packets that hit the boundary of the PESs. The pump pulse is assumed to be a δ pulse projecting the ground state wave function from V_0 to V_1. The time delay is defined to be zero at ≈7.2 fs such that the pulse with FWHM of 5.29 fs used in Fig. 3(b) vanishes at the beginning of the propagation.

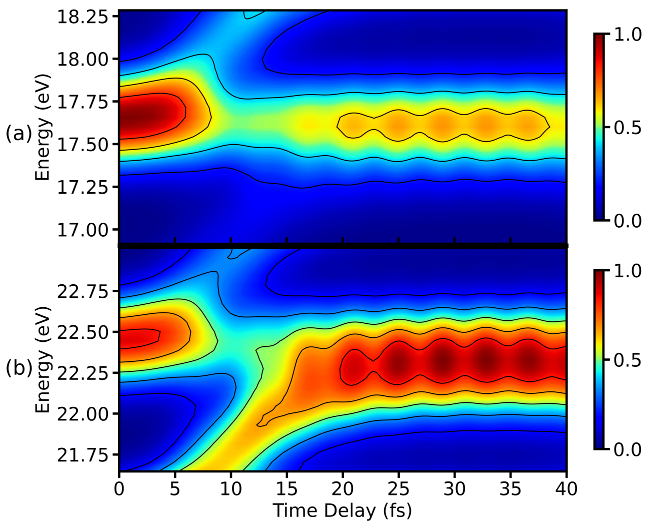

Figure S5. The TRPES spectrum corresponding to (a) H_{43}, and (b) H_{51} ionizing V_1 states are shown here for APT with ω_I=0.6 eV.

IV. CLOSE UP OF FIG. 4(B)

The TRPES spectra for two different high-harmonics for attosecond pulse train (APT) with IR pulse frequency of 0.6 eV are shown in Fig. S5. The spectrum shown in Fig. S5(a) corresponds to the harmonic H_{43} ionizing state V_1, which overlaps with the signal corresponding to H_{41} ionizing state V_0. Since the intensity of H_{43} is higher compared to H_{41}, and the diabatic population in V_0 state is less compared to V_1 state after conical intersection (CI), the spectrum mainly shows the signal from V_1 state. This allows for a precise observation of V_1 state along with hints of electronic coherence appearing. For the spectrum in Fig. S5(b), the signal corresponding to H_{51} ionizing state V_1 is somewhat less intense than the overlapping signal corresponding to H_{49} ionizing state V_0. This comes from the fact that harmonic H_{51} has weaker amplitude than H_{49}. This results in a wider spectral range of the signal after the CI, making it difficult to observe the electronic states more accurately, but shows rather strong electronic coherence.

[1] Bennett, K.; Kowalewski, M.; Mukamel, S. Nonadiabatic dynamics may be probed through electronic coherence in time-resolved photoelectron spectroscopy. *J Chem. Theory Comput.* **2016**, *12*, 740–752.

[2] Mukamel, S.; Rahav, S. *Advances in atomic, molecular, and optical physics*; Elsevier, 2010; Vol. 59; pp 223–263.

[3] Kowalewski, M.; Mukamel, S. Stimulated Raman signals at conical intersections: Ab initio surface hopping simulation protocol with direct propagation of the nuclear wave function. *J Chem. Phys.* **2015**, *143*, 044117.

[4] Arnoldi, W. E. The principle of minimized iterations in the solution of the matrix eigenvalue problem. *Quarterly of applied mathematics* **1951**, *9*, 17–29.

[5] Nissen, A.; Karlsson, H. O.; Kreiss, G. A perfectly matched layer applied to a reactive scattering problem. *J. Chem. Phys.* **2010**, *133*, 054306.
